# Identification of QTLs Associated with Callogenesis and Embryogenesis in Oil Palm Using Genetic Linkage Maps Improved with SSR Markers

**DOI:** 10.1371/journal.pone.0053076

**Published:** 2013-01-29

**Authors:** Ngoot-Chin Ting, Johannes Jansen, Jayanthi Nagappan, Zamzuri Ishak, Cheuk-Weng Chin, Soon-Guan Tan, Suan-Choo Cheah, Rajinder Singh

**Affiliations:** 1 Advanced Biotechnology and Breeding Centre, Malaysian Palm Oil Board, Kajang, Selangor, Malaysia; 2 Biometris, Wageningen University and Research Centre, AC Wageningen, The Netherlands; 3 Federal Land Development Authority Malaysia Biotechnology Centre, Federal Land Development Authority Malaysia Agricultural Services Sdn. Bhd., Kuala Lumpur, Malaysia; 4 Department of Cell and Molecular Biology, Universiti Putra Malaysia, Serdang, Selangor, Malaysia; University of Nottingham, United Kingdom

## Abstract

Clonal reproduction of oil palm by means of tissue culture is a very inefficient process. Tissue culturability is known to be genotype dependent with some genotypes being more amenable to tissue culture than others. In this study, genetic linkage maps enriched with simple sequence repeat (SSR) markers were developed for *dura* (ENL48) and *pisifera* (ML161), the two fruit forms of oil palm, *Elaeis guineensis*. The SSR markers were mapped onto earlier reported parental maps based on amplified fragment length polymorphism (AFLP) and restriction fragment length polymorphism (RFLP) markers. The new linkage map of ENL48 contains 148 markers (33 AFLPs, 38 RFLPs and 77 SSRs) in 23 linkage groups (LGs), covering a total map length of 798.0 cM. The ML161 map contains 240 markers (50 AFLPs, 71 RFLPs and 119 SSRs) in 24 LGs covering a total of 1,328.1 cM. Using the improved maps, two quantitative trait loci (QTLs) associated with tissue culturability were identified each for callusing rate and embryogenesis rate. A QTL for callogenesis was identified in LGD4b of ENL48 and explained 17.5% of the phenotypic variation. For embryogenesis rate, a QTL was detected on LGP16b in ML161 and explained 20.1% of the variation. This study is the first attempt to identify QTL associated with tissue culture amenity in oil palm which is an important step towards understanding the molecular processes underlying clonal regeneration of oil palm.

## Introduction

Tissue-cultured oil palm clones are in high demand because of their greater uniformity and higher yields compared to conventional seedling material [1]. Current commercial seedling material consists of hybrids, referred to as *tenera*, that result from crossing *dura* and *pisifera* palms, the two fruit forms of the African oil palm (*Elaeis guineensis* Jacq.). A large range of variation of up to 30.0% from the mean yield can be observed in *tenera* [2]. On the other hand, the best clones have been reported to yield at least 30.0% more than seedling populations [3], although admittedly there is a problem choosing representative seedlings standard for clonal trials [4]. Although the projected yield increases of up to 30.0% for clonal palms have met with some skepticism, the oil palm industry is confident that eventually the use of clonal planting material will lead to the “next wave” of yield improvement. For this reason in Malaysia twelve oil palm tissue culture laboratories produce annually over two million clonal palms (or ramets), mostly for evaluation within their own organizations [5].

However, clonal reproduction of oil palm is beset by a host of challenges and thus requires further improvements to cope with an ever increasing demand. Too long a period in culture can give rise to abnormal ramets, the causes of which are still being investigated. This *per se* can be overcome by merely culturing more palms with more lines making up for the shorter runs. But, herein lies the second, perhaps more insidious problem – oil palm tissue culture is a very inefficient process with, on average, over 80.0% of the cultures failing to generate plants [6]. The reasons for this are not known but similar results are obtained with the tissue culture of other major economic crops, like rice, tobacco, potato and tomato [7]. Hence, the efficiency of tissue culture has to be improved if the Malaysian oil palm industry wants to realize its target of producing 40 million ramets by 2017 [8].

Evidence exist that tissue culturability of oil palm has a genetic basis with some genotypes being more amenable to tissue culture than others [9]. The question is whether genotypes with improved tissue culturability can be identified. In several plant species, the genomic loci affecting tissue culturability have been mapped as quantitative trait loci (QTL) on genetic linkage maps. QTL responsible for tissue culture amenity have been identified in rice [10], wheat [11] and barley [12]. This demonstrates the potential of this approach for identifying markers associated with tissue culture response. However, to date no QTL for tissue culturability has been reported for oil palm.

In this study, both genomic and EST-SSR markers were generated and mapped to the Ulu Remis Deli *dura* (ENL48) and Yangambi *pisifera* (ML161) genetic maps reported earlier [13]. The use of common SSR markers allowed the present linkage maps to be linked to the oil palm reference map [14]. This also allowed standardized labeling and orientation of linkage groups with the reference map. This study is the first attempt to identify QTL associated with tissue culture amenity in oil palm. This report also discusses the potential application of the markers linked to the QTL for tissue culturability to improve the efficiency of clonal propagation in oil palm.

## Materials and Methods

### Mapping Population

The mapping population (P2) consisted of 87 F_1_ palms obtained from a cross between Ulu Remis Deli *dura* (ENL48) and Yangambi *pisifera* (ML161) [13] grown at Kota Gelanggi, Malaysia. The two parental palms were not cultured due to the long recovery period anticipated after tissue culture, which would have interfered with the on-going breeding program.

### Initiation of Calli and Embryoids

**Figure 1:**
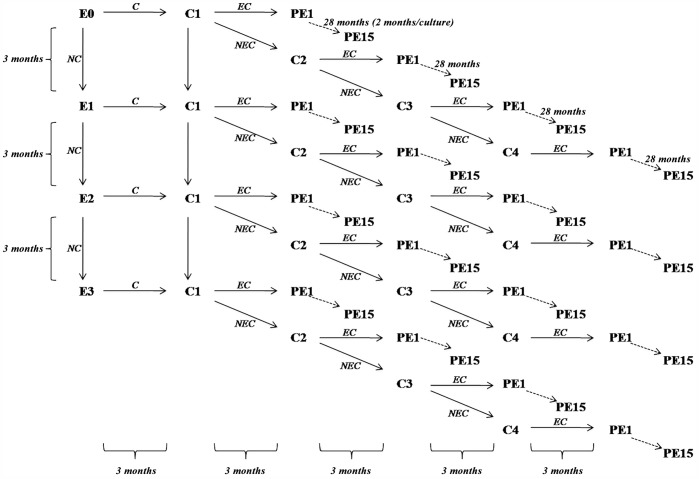
General workflow of oil palm tissue culture. Explant (E0) is cultured to form callus (*C*) which is transferred to a new medium (C1) to form embryoids. Cultures not forming callus (*NC*) are transferred to a fresh medium (**E1**–**E3**) and undergo the same process again. Embryoids (*EC*) generated from **C1** proceed to polyembryoid culture (**PE1**–**PE15**) for the regeneration of plantlets. Callus cultures that fail to generate embryoids (*NEC*) are transferred to a fresh medium (**C2**–**C4**) and undergo the same process again.

The general flow of the tissue culture process is described in Figure 1. Tissue culture was carried out by the Malaysian Palm Oil Board (MPOB) and seven collaborating laboratories (listed in the Acknowledgements) with each culturing a number of palms to its capacity using standardized procedures. Each palm in the mapping population was sampled for tissue culture by carefully harvesting the unopened spear leaves (leaf cabbage) as shown in Figure S1.

Both ends of the cabbage and its outer layers were removed except the petioles of frond number 0. All the surfaces were swabbed with absolute alcohol. This was followed by a longitudinal cut to disclose the internal fronds (fronds −3 to −7 or lower) comprising stacks of young leaflets. In order to avoid contamination, about 10 cm at the distal ends of leaflets were discarded and the remaining leaflets were cut into 12 segments of approximately 1.5 cm width. The explants were sterilized in the following steps: i. immersion in a freshly prepared calcium hypochlorite solution (45 g/l) at room temperature for 20 min, ii. rinsing with sterile-distilled water for 10 sec and, iii. dipping in 30 g/l sterile glucose solution before culturing on the modified medium of Murashige and Skoog [15] containing one of two concentrations of alpha-naphthalene acetic acid (NAA), hereinafter referred to as Treatments 1 and 2.

In Treatment 1, explants were inoculated at 28±2°C under continuous darkness for three months in 5 mg/l NAA (**E0**). Explants that did not form callus (*NC*) were transferred to a fresh medium similar to E0 and undergo the same process at **E1**, **E2** and **E3**. The resulting calli were transferred to a new medium containing 5 mg/l NAA (**C1**) to form embryoids (*EC*). This process was followed by polyembryoid cultures (**PE1**–**PE15**) with 0.1 mg/l NAA. Each PE subculture took two months under 12 hr photoperiod. Callus culture that failed to generate embryoid (*NEC*) was transferred to a fresh medium to undergo the same process again (**C2**–**C4**). For Treatment 2, 10 mg/l NAA was used for the cultures of explants and calli (**E0**–**E3** and **C1**–**C4**), followed by 0.5 mg/l NAA in **PE1**–**PE3** and 0.1 mg/l NAA in **PE4**–**PE15**.

Over a period of two years, the callusing rate (CR) and embryogenesis rate (ER) were determined. CR and ER were measured as: CR = (total number of calli formed from **E0** to **E3**/total number of clean cultures)×100.0%; ER = (total number of embryoid lines formed from **C1** to **C4**/total number of calli formed)×100.0%. The measurements were labeled as CR1 and ER1 and, CR2 and ER2, for Treatment 1 and 2, respectively.

### DNA Extraction

Genomic DNA was extracted from the spear leaves (stored at −80°C) of the 87 progenies and the two parental palms using the modified CTAB method [16]. The DNA concentration and purity were measured using a UV/VIS Spectrometer (Perkin-Elmer Lambda Bio 2.2). For SSR analysis, DNA was prepared at 50 ng/ul in TE (pH 8.0) buffer.

### SSR Analysis

In this study, SSR primers were mainly obtained from the oil palm SSR collection of MPOB. Additional genomic SSR primer sequences were downloaded from the TropGENE database (http://tropgenedb.cirad.fr/html/oilpalm_Marker.html) and labeled as mEgCIR. MPOB in-house SSRs were developed from the oil palm ESTs and genomic sequences reported by [17]–[20]. SSRs developed from *E. guineensis* expressed sequence tags (ESTs), *E. guineensis* genomic sequences, *E. oleifera* genomic sequences and interspecific hybrid (*E. guineensis*×*E. oleifera)* genomic sequences were labeled sEg, sMg, sMo and sMh, respectively.

Screening and genotyping of polymorphic SSRs were carried out as described by [19]. In addition, an ABI3100 genetic analyzer (Applied Biosystems, UK) was used to accelerate the genotyping process using M13-tailed primers as described by [21]. A 19-bp M13 sequence (CACGACGTTGTAAAACGAC) was attached to each of the forward primers (Fwd 5′-M13) and the fluorescent dye (HEX−/6-FAM−/NED-M13). PCR was carried out in a 10.0 ul volume containing 100 ng DNA, 1×PCR buffer (NEB, USA), 2 mM of each dNTP (NEB, USA), 2.5 uM of each primer (Fwd 5′-M13, reverse unlabelled primer and dye-M13 primer) and 0.5 U *Taq* DNA polymerase (NEB, USA). PCR was carried out as described by [19]. A maximum of three PCR products were each labeled with HEX, 6-FAM and NED and multiplexed at a ratio of 1∶1:2. Two-ul of the multiplexed mix was denatured in 7.84 ul Hi-Di™ Formamide (Applied Biosystems, UK) and 0.16 ul GeneScan™ 400HD ROX® Size Standard (Applied Biosystems, UK). The denatured sample was then fragmented and size-called on the ABI3100 genetic analyzer.

Genotype data generated from the SSR analysis were scored based on the segregation profiles 1, 5, 8 and 9 in [14] which are illustrated in Table S1. In profile 1, polymorphism of the locus was observed in either one of the parents. The heterozygous and homozygous genotypes were scored as *lm* and *ll* for allele segregating in the ENL48 parent and, *np* and *nn* for the *pisifera* parent. The ratio for genotypes *lm*:*ll* and *nn*:*np* are expected to follow the 1∶1 segregation ratio. In profile 5, the Mendelian segregation ratio of 1∶2:1 (for combination *hh*:*hk*:*kk*) is expected when two common alleles (*h* and *k*) are segregating in both parents. Profile 8 shows three co-segregating alleles with one common allele (scored as *e*) in both the parents and two different alleles (scored as *f* and *g*). The segregation of these alleles *ee*:*ef*:*eg*:*fg* is expected to be in 1∶1:1∶1 ratio. In the configuration where there are four co-segregating alleles (profile 9), two different alleles are segregating in each parent and they were scored as *a* and *b* in parent ENL48 and *c* and *d* in parent ML161. The genotypes *ac*:*bc*:*ad*:*bd* are also expected to segregate according to a 1∶1:1∶1 ratio.

### RFLP Analysis

RFLP analysis was carried out according to [22] with most of the marker data generated by [13], [23]. The markers were named after different tissue types from which the cDNA probes were obtained. The nomenclatures used were: CA/CB (non-embryogenic callus), CEO (embryogenic callus), EA/EO (proliferating embryoid), FDA/FDB/SFB (inflorescence), G/GT (young etiolated seedling), K/KD/KT (kernel), M/ME/MET/MT (mesocarp) and RD (root). Scoring of the RFLP marker was similar to SSR.

### AFLP Analysis

AFLP markers were generated using three restriction enzyme-combinations: *Eco*RI/*Mse*I, *Pst*I/*Mse*I and *Taq*I/*Hind*III as described by [13], [23]. The marker nomenclature represents the selective primer-pair followed by size of the observed fragment. Data was scored for polymorphic fragments as in profile 1 (Table S1).

### Genotypic Data Analysis and Construction of the Parental Linkage Maps

The SSR data were incorporated into the previous parental data sets consisting of RFLP and AFLP markers. Chi-square analysis was performed to determine markers with distorted segregation at several levels from P<0.0001 to <0.1. Markers showing distorted segregation and missing data were excluded as per the criteria of [24]. In this study, the mapping strategy was to examine marker data in a systematic manner, thereby removing problematic markers at every step of the map construction process.

Linkage analysis was carried out separately for ENL48 and ML161 using JoinMap® 4.0 [25]. All markers (except those with segregation type <hkxhk>) were re-coded to the double-haploid (DH1) format as described by [26], which is equivalent to the double pseudo-testcross approach [27]. Subsequently, the re-coded markers were grouped using a recombination frequency threshold of 0.2 and the linkage phases of the markers were determined. For each parent, a basic map was constructed using the maximum likelihood method. Markers with a nearest neighbor stress (N.N. Stress) value greater than 4 (cM) were discarded from further analysis.

Markers of segregation type <hkxhk> were subsequently included with those mapped in the basic maps. The dataset (now including the <hkxhk> markers) was re-analyzed using the regression mapping function in JoinMap® 4.0. Markers were grouped using a recombination frequency threshold of 0.2. The recombination frequencies between markers were transformed into map distances in centiMorgan (cM) using the Haldane mapping function. On each linkage group, the contribution of each marker to the average goodness-of-fit (mean Chi-square) and nearest neighbor fit (N.N. Fit) was inspected to confirm its most likely position in order to get the best possible map. In addition, stability of the marker order on every linkage group was checked by comparing with the parental maps (generated earlier using DH1 format) using MapChart 2.2 [28]. The <hkxhk> type markers that caused a change in order were discarded.

### Statistical Analysis

The CR was transformed using a log-transformation {*ln* (CR +0.2)}, subsequently denoted as LnCR. Approximately half of the observed ER were equal to zero. Therefore, two transformations were used: (1) a transformation into a binary variable, denoted as binER, with values: 0 if ER = 0 and 1 if ER >0, (2) a transformation into an ordinal variable, denoted as ordER, with three values: 0 if ER = 0, 1 if 0< ER ≤1 and 2 if ER >1.

The following analysis was made based on the fact that palms were assigned randomly to eight laboratories. Differences between laboratories and treatments were removed by using a mixed model, *y_ijk_* = *µ*+*l_i_*+*t_j_*+*p_ik_*+*e_ijk_*, in which *y_ijk_* is the observation on palm *k* ( = 1…*n_j_*) assigned to laboratory *i* ( = 1…8) with treatment *j* ( = 1, 2), *l_i_* is the fixed effect of laboratory *i*, *t_j_* is the fixed effect of treatment *j*, *p_ik_* is the random effect of palm *k* within laboratory *i* (with zero mean and variance *σ*_p_^2^) and *e_ijk_* is a residual effect (with zero mean and variance *σ*_e_^2^). Parameter estimation was carried out using the REML facilities in GenStat 14 [29]. Predictions of the random effects *p_ik_*, denoted as PLnCR, PbinER and PordER, respectively, were subjected to QTL analysis. The coefficient of determination was calculated as *σ*_p_^2/^(*σ*_p_^2^+ *σ*_e_^2^), with parameters replaced with estimates; the coefficient of determination is a measure of resemblance of the observations under Treatments 1 and 2.

### Detection of Quantitative Trait Loci (QTL)

Detection of QTL was carried out using the GenStat QTL library [29]. The traits PLnCR and PbinER, and PLnCR and PordER, were subjected to a two-trait QTL analysis. Tests for significance of QTL were only carried out at marker positions. For determining the significance threshold, the method of Li and Ji [30] was used with a genome wide significance level of 95.0%. The final selection of QTL was obtained after MQM mapping and backward elimination of putative QTL.

## Results

### Phenotyping and Evaluation of Callogenesis and Embryogenesis Data

In the tissue culture of each palm, approximately equal numbers of explants were replicated for culture on Treatments 1 and 2. The exceptions were palm number 87, which had a difference of 92 explants between the two replicates and palm 75 which was unfit for sampling during the period of the research program. The numbers of actual explants for each palm ranged from 412–1,158 depending on the numbers of internal fronds available for tissue culture.

A high variation of CR was observed in Treatment 1∶0–47.2% and in Treatment 2∶0.14–41.7%. For ER which was calculated as the total number of embryoids formed from calli ranged from 0–21.1% in Treatment 1 and 0–45.2% in Treatment 2 (Figure S2). The determination coefficient for CR was equal to 0.94, showing a high level of resemblance of the values of the two treatments. For ER the determination coefficient was equal to 0.48 for the binary scoring (binER) and 0.51 for the ordinal scoring (ordER) with three categories, showing an intermediate level of resemblance.

In this study, the callusing and embryogenesis data were obtained by eight different laboratories, with two different treatments. Therefore, further analysis was carried out to determine if these experimental variables affected the phenotypic data. Table 1 shows the results of the mixed model analysis. This table shows that large and significant differences occur between laboratories. However, no significant differences between the treatments were found. Predictions of LnCR, binER and ordER for individual palms (after removing the effects of treatments and laboratories) obtained from the mixed model analysis were used for QTL analysis. The predictions were denoted as PLnCR, PbinER and PordER, respectively. Table 2 summarizes the means of callusing (LnCR) and embryogenesis rates (binER and ordER).

**Table 1:** Estimates of variation components and effects of laboratories and treatments on LnCR, binER and ordER.

	LnCR		binER		ordER	
Random effects:	Component	S.E	Component	S.E	Component	S.E
Labs and palms	0.963	0.160	0.108	0.028	0.382	0.096
Residuals	0.060	0.009	0.118	0.018	0.369	0.057
Fixed effects:	Wald statistic	p-value	Wald statistic	p-value	Wald statistic	p-value
Labs	35.41	<0.001	17.45	0.023	18.01	0.019
Treatments	0.06	0.800	0.80	0.374	1.29	0.259

**Table 2:** Means of callusing (LnCR) and embryogenesis (binER and ordER) observed for samples tissue cultured by the different laboratories.

Labs	No. ofpalmscultured	LnCR ± S.E	binER ± S.E	ordER ± S.E
1	33	1.032±0.137	0.318±0.062	0.546±0.113
2	8	2.426±0.336	0.375±0.140	0.563±0.258
3	8	1.617±0.336	0.125±0.140	0.250±0.258
4	2	2.409±0.697	0.250±0.287	0.500±0.528
5	8	1.541±0.336	0.813±0.140	1.563±0.258
6	8	3.017±0.336	0.625±0.140	1.063±0.258
7	11	1.732±0.281	0.500±0.118	0.864±0.217
8	7	2.113±0.361	0.571±0.150	0.929±0.276

### SSR Data

In this study, a total of 342 polymorphic SSRs were generated from the collection of sequences at MPOB (sEg, sMg, sMo and sMh) and the public database (mEgCIR). The SSRs were scored for polymorphisms based on the profiles by [14] where 252 (81 in ENL48, 171 in ML161), 20, 49 and 21 SSRs were scored as having profiles 1, 5, 8 and 9, respectively. In ENL48, the polymorphic SSRs comprised of 55 mEgCIR, 27 sEg, 50 sMg and 39 sMo whereas in ML161, the 265 SSRs comprised of 86 mEgCIR, 42 sEg, 75 sMg, 61 sMo and 1 sMh. Of these, the majority of SSRs segregated in either one of the parents and 26.3% were polymorphic in both ENL48 and ML161.

Subsequently, the SSR data were combined with the existing RFLP and AFLP data sets to generate the genetic map for the parental palms. Previously, we had generated 152 AFLPs and 102 RFLPs for ENL48 and, 272 AFLPs and 165 RFLPs for ML161 inclusive of data reported in [13]. The numbers of each marker types used for map construction is summarized in Table S2.

### The ENL48 and ML161parental Maps

With the addition of SSR markers, the number of markers in ENL48 increased to 425 (152 AFLPs, 102 RFLPs and 171 SSRs) and 702 (272 AFLPs, 165 RFLPs and 265 SSRs) in ML161. In ENL48, 55 markers (49 AFLPs and 6 RFLPs) with ≥10.0% missing data and 12 markers (9 AFLPs, 1 RFLP and 2 SSRs) with severe segregation distortion (p<0.0001) were excluded from further analysis. A majority of the remaining skewed markers showed distorted segregations at p<0.05–0.1 and less than 10.0% at p<0.0005–0.01. Similar criteria were also used to examine the ML161 data set, where 94 markers were excluded (83 AFLPs, 6 RFLPs and 5 SSRs). After removing the severely distorted markers at p<0.0001, the percentage of distortion observed in ML161 was about 2.0%, considerably lower than that (18.0%) in ENL48.

**Figure 2:**
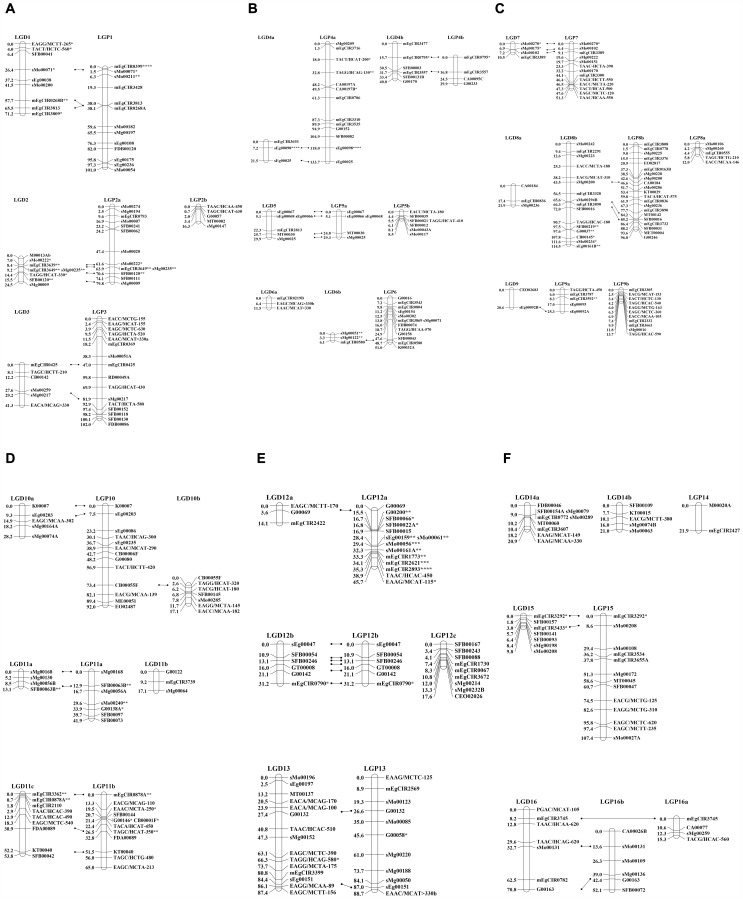
Alignment of the ENL48 (left) and ML161 (right) maps using co-segregating markers. Markers showing distorted segregation are indicated by asterisk (*) representing significance at p<0.1; (**) p<0.05; (***) p<0.01; (****) p<0.05 and; (******) p<0.0005.

Finally, the data set used for construction of the ENL48 linkage map consisted of 94 AFLPs, 95 RFLPs and 169 SSRs. Of the 358 markers analyzed, 330 were assembled into 23 groups. In order to determine the best position for every marker in a linkage group, the markers contributing to insufficient linkages were also determined and removed. The final map consisted of 148 markers (33 AFLPs, 38 RFLPs and 77 SSRs) in 23 groups (Figure 2). The sequences of the SSR primers and the RFLP clones mapped in this study have been deposited into public databases with their accession numbers shown in Tables 3 and 4.

**Table 3:** SSR markers mapped on both the ENL48 and ML161 parental maps and their accession numbers.

No.	SSR locus	Linkage group		TA (°C)	SSR motif	Accession no.	Putative ID [organism] Blast search was carried out on 12^th^ Oct2012
		ENL48	ML161				
1.	sEg00025	LGD4a	LGP4a	53	(TTA)_10_	EY397492^c^	No significant similarity
2.	sEg00038	LGD1		52	(AAT)_9_	Pr009947960^d^	No significant similarity
3.	sEg00047	LGD12b	LGP12b	56	(AT)_12_	EY400727^c^	Predicted: uncharacterized protein LOC100243686 [*Vitis vinifera*]
4.	sEg00066	LGD5	LGP5a	52	(AT)_8_	EY403542^c^	No significant similarity
5.	sEg00067	LGD5	LGP5a	52	(TGTA)_6_	EY404537^c^	No significant similarity
6.	sEg00068	LGD5	LGP5a	53	(AT)_8_	EY404017^c^	No significant similarity
7.	sEg00086		LGP10	57	(ATAC)_10_	EY407048^c^	Predicted: Putative pterin-4-alpha-carbinolamine dehydratase isoform 1 [*Vitis vinifera*]
8.	sEg00092^a^	LGD9	LGP9a	52	(TATG)_6_	EY407741^c^	No significant similarity
9.	sEg00095		LGP9a	52	(TATG)_5_	EY405343^c^	No significant similarity
10.	sEg00098	LGD4a	LGP4a	52	(GGT)_6_	EY405527^c^	Developmentally regulated GTP-binding protein, putative [*Ricinus communis*]
11.	sEg00108		LGP1	57	(CGG)_8_	EY408074^c^	Histone deacetylase [*Populus trichocarpa*]
12.	sEg00151	LGD13	LGP13	57	(CAG)_8_	EY411661^c^	Transcription factor [*Lycoris longituba*]
13.	sEg00154		LGP6	57	(CAG)_5_	EY410356^c^	Predicted: Transcription factor bHLH96-like [*Vitis vinifera*]
14.	sEg00159		LGP12a	57	(AT)_9_	EY408671^c^	TGA transcription factor [*Medicago truncatula*]
15.	sEg00161	LGD8b		57	(AT)_15_	EY410342^c^	Cytosolic aldehyde dehydrogenase RF2C [*Zea mays*]
16.	sEg00175		LGP1	57	(CT)_7_gttttttcccctttgttccctggtgaga(TTG)_6_	EY413618^c^	Uncharacterized protein LOC100502350 precursor [*Zea mays*]
17.	sEg00197^b^	LGD13		59	(GA)_10_	EL684358^c^	Predicted: Uncharacterized protein LOC100828466 [*Brachypodium distachyon*]
18.	sEg00203^b^	LGD10a	LGP10	58	(CT)_7_	EL595513^c^	Hypothetical protein SORBIDRAFT_07g019420 [*Sorghum bicolor*]
19.	sEg00235		LGP10	51	(CT)_9_	EY409185^c^	Putative oxalyl-CoA decarboxylase [*Vitis vinifera*]
20.	sEg00236		LGP1	55	(CT)_7_	EY413618^c^	Hypothetical protein SORBIDRAFT_10g023220 [*Sorghum bicolor*]
21.	sMg00009	LGD2	LGP2a	52	(AT)_13_	Pr010615860^d^	No significant similarity
22.	sMg00016		LGP9b	52	(GA)_13_	Pr010615861^d^	No significant similarity
23.	sMg00025	LGD5	LGP5a	52	(TC)_11_	Pr010615864^d^	No significant similarity
24.	sMg00050		LGP13	50	(TA)_17_	Pr010615868^d^	No significant similarity
25.	sMg00051	LGD6b		52	(CT)_7_(AGAA)_6_	Pr010615869^d^	No significant similarity
26.	sMg00056^a^	LGD11a	LGP11a	53	(CT)_18_	Pr010615871^d^	No significant similarity
27.	sMg00064	LGD11b		52	(GA)_10_	Pr010882584^d^	No significant similarity
28.	sMg00071		LGP6	54	(GAA)_8_GGAG(GCT)_13_	Pr010615877^d^	No significant similarity
29.	sMg00074^a^	LGD10a, D14b		52	(AGG)_9_AGCCCAGCCCTCGTCCACCTTTT(GCC)_5_	Pr010615878^d^	Predicted: *Vitis vinifera* uncharacterized LOC100260255 (LOC100260255), mRNA. [*Vitis vinifera*]
30.	sMg00079	LGD14a		54	(TG)_7_(AG)_11_	Pr010615879^d^	No significant similarity
31.	sMg00122	LGD6b		54	(AT)_18_	Pr010615882^d^	No significant similarity
32.	sMg00130	LGD11a		52	(TA)_14_	Pr010615883^d^	No significant similarity
33.	sMg00136		LGP16b	56	(AG)_11_	Pr010615884^d^	No significant similarity
34.	sMg00147		LGP2b	56	(AT)_11_	Pr010615886^d^	No significant similarity
35.	sMg00152	LGD13		54	(AT)_13_	Pr010615887^d^	No significant similarity
36.	sMg00164	LGD10a		55	(TA)_12_	Pr010615889^d^	No significant similarity
37.	sMg00168	LGD11a	LGP11a	55	(CT)_11_	Pr010615890^d^	No significant similarity
38.	sMg00172		LGP15	56	(CT)_14_	Pr010615891^d^	Predicted protein [*Populus trichocarpa*]
39.	sMg00175	LGD7		54	(CGG)_10_	Pr010615892^d^	*Camellia sinensis* clone U10BCDNA 1304 50S ribosomal protein L29 mRNA, complete cds [*Camellia sinensis*]
40.	sMg00188		LGP13	52	(ACCG)_8_	Pr010615894^d^	No significant similarity
41.	sMg00194		LGP2a	54	(TA)_26_	Pr010615897^d^	No significant similarity
42.	sMg00197		LGP1	56	(AG)_15_	Pr010615898^d^	No significant similarity
43.	sMg00198	LGD15		56	(AG)_14_	Pr010615899^d^	No significant similarity
44.	sMg00200	LGD8b	LGP8b	60	(CT)_18_	Pr010615900^d^	No significant similarity
45.	sMg00209		LGP4a	54	(GA)_14_	Pr010615903^d^	No significant similarity
46.	sMg00214		LGP12c	52	(AT)_14_	Pr010615905^d^	No significant similarity
47.	sMg00217	LGD3	LGP3	54	(GA)_16_	Pr010615907^d^	No significant similarity
48.	sMg00220		LGP13	52	(AT)_19_	Pr010615909^d^	No significant similarity
49.	sMg00222		LGP7	50	(AG)_20_	Pr010615910^d^	No significant similarity
50.	sMg00223	LGD8b		56	(GA)_14_	Pr010615911^d^	No significant similarity
51.	sMg00225		LGP8b	56	(TC)_14_	Pr010615912^d^	No significant similarity
52.	sMg00228		LGP8b	54	(AT)_25_	Pr010615913^d^	No significant similarity
53.	sMg00232		LGP12c	54	(GA)_15_	Pr010615915^d^	No significant similarity
54.	sMg00235	LGD2	LGP2a	58	(GA)_15_	Pr010615916^d^	Predicted: *Vitis vinifera* hydroxysteroid 11-beta-dehydrogenase 1-like protein (LOC100260124), mRNA [*Vitis vinifera*]
55.	sMg00236	LGD8a	LGP8b	56	(TC)_18_	Pr010615917^d^	No significant similarity
56.	sMg00259		LGP16a	57	(C)_11_	Pr010615923^d^	No significant similarity
57.	sMg00260		LGP8a	57	(CTG)_5_	Pr010615924^d^	Predicted: *Glycine max* DELLA protein DWARF8-like (LOC100805968), mRNA [*Glycine max*]
58.	sMo00007		LGP2a	50	(TA)_12_	Pr010615926^d^	No significant similarity
59.	sMo00020		LGP2a	58	(AG)_15_	Pr009947964^d^	No significant similarity
60.	sMo00027		LGP15	50	(TC)_14_	Pr009947965^d^	No significant similarity
61.	sMo00043		LGP5b	50	(AG)_14_	Pr010615928^d^	No significant similarity
62.	sMo00051		LGP3	54	(TA)_20_	Pr010615929^d^	No significant similarity
63.	sMo00054		LGP1	54	(TA)_12_	Pr010615930^d^	No significant similarity
64.	sMo00056		LGP12a	54	(CT)_11_	Pr010615931^d^	No significant similarity
65.	sMo00061		LGP12a	56	(CT)_12_	Pr010615932^d^	No significant similarity
66.	sMo00063	LGD14b		54	(GA)_12_	Pr010615933^d^	No significant similarity
67.	sMo00071	LGD1	LGP1	56	(AG)_22_	Pr010615934^d^	No significant similarity
68.	sMo00085		LGP13	56	(TC)_12_	Pr010882585^d^	cDNA clone:OSIGCRA119H18, full insert sequence [*Oryza sativa* indica cultivar-group]
69.	sMo00102	LGD7	LGP7	53	(AG)_11_	Pr010615939^d^	No significant similarity
70.	sMo00106		LGP8a	52	(CT)_20_	Pr010615940^d^	No significant similarity
71.	sMo00108		LGP15	53	(AT)_19_	Pr010882586^d^	Predicted: *Vitis vinifera* uncharacterized LOC100263245 (LOC100263245), mRNA [*Vitis vinifera*]
72.	sMo00109		LGP16b	56	(TA)_23_	Pr010882587^d^	No significant similarity
73.	sMo00117		LGP5b	54	(AG)_14_	Pr010615941^d^	No significant similarity
74.	sMo00123		LGP13	54	(TC)_12_	Pr010882588^d^	No significant similarity
75.	sMo00131	LGD16	LGP16b	54	(TTA)_19_	Pr010615943^d^	No significant similarity
76.	sMo00151		LGP7	50	(TG)_6_tc(TA)_10_a(AATAT)_5_	Pr010615945^d^	No significant similarity
77.	sMo00161		LGP12a	54	(TG)_8_(AG)_8_	Pr010317032^d^	No significant similarity
78.	sMo00170		LGP7	53	(GA)_17_	Pr010615948^d^	No significant similarity
79.	sMo00182		LGP1	58	(CTC)_5_gtctacctccgcctccaccgccaccgcagagccatccttctcttctgcacct(TCC)_5_	Pr010615949^d^	No significant similarity
80.	sMo00196	LGD13		56	(ACAA)_8_(ACAT)10(AT)_10_	Pr010615950^d^	No significant similarity
81.	sMo00200	LGD1		57	(ATAC)_6_(AT)_18_	Pr010615951^d^	No significant similarity
82.	sMo00208	LGD15	LGP15	58	(TC)_10_	Pr010615952^d^	No significant similarity
83.	sMo00211		LGP1	57	(AC)_7_	Pr010615953^d^	No significant similarity
84.	sMo00222	LGD2	LGP2a	57	(CT)_8_	Pr010615956^d^	Predicted: *Vitis vinifera* hydroxysteroid 11-beta-dehydrogenase 1-like protein (LOC100260124), mRNA [*Vitis vinifera*]
85.	sMo00234	LGD8b		57	(TC)_8_	Pr010615957^d^	*Phoenix dactylifera* mitochondrion, complete genome [*Phoenix dactylifera*]
86.	sMo00240		LGP11a	57	(GA)_8_	Pr010615958^d^	Predicted: *Vitis vinifera* dynamin-related protein 1E-like,transcript variant 1 (LOC100266825), mRNA. [*Vitis vinifera*]
87.	sMo00242	LGD8b		51	(TC)_11_	Pr010615959^d^	No significant similarity
88.	sMo00259	LGD3		56	(AGA)_5_	Pr010615961^d^	No significant similarity
89.	sMo00270	LGD7	LGP7	57	(TTC)_6_	Pr010615963^d^	No significant similarity
90.	sMo00274		LGP2a	58	(AGA)_5_	Pr010882589^d^	No significant similarity
91.	sMo00285	LGD10b		56	(ACC)_6_	Pr010615964^d^	No significant similarity
92.	sMo00286		LGP8b	57	(CGG)_8_	Pr010615965^d^	No significant similarity
93.	sMo00289	LGD14a		58	(TGT)_8_	Pr010615966^d^	No significant similarity
94.	sMo00294	LGD8b		57	(ACAT)_8_	Pr010615968^d^	No significant similarity
95.	sMo00302		LGP6	56	(AG)_7_	Pr010615970^d^	No significant similarity

Putative IDs were deduced for the SSR-containing sequences by comparing to the non-redundant protein database (Blastx for EST sequences) and nucleotide database of GenBank (tBlastx for genomic sequences). A threshold score of >80 was used to assign significant similarity.

a Two SSR markers were mapped.

b SSRs developed from oil palm sequences from NCBI GenBank.

c Accession numbers of NCBI GenBank.

d Probe Unique Identifiers (PUIDs) of NCBI Probe Database.

**Table 4:** RFLP markers mapped on both the ENL48 and ML161 parental maps with their GenBank accession numbers.

No.	RFLP locus	Linkage group		Accession no.	Putative ID [organism] Blast search was carried out on 12^th^ Oct 2012
		ENL48	ML161		
1	CA00026B		LGP16b	EY396203	Aquaporin [*Elaeis guineensis*]
2	CA00077		LGP16a	JK629436	Hox12, partial [*Oryza sativa* Indica Group]
3	CA00095		LGP4b	JK629437	Ubiquitin carrier protein [*Elaeis guineensis*]
4	CA00184	LGD8a	LGP8b	GH159163	Cyclin d, putative [*Ricinus communis*]
5	CA00197		LGP4a	EY396360^a^	Predicted: uncharacterized protein LOC100249262 [*Vitis vinifera*]
6	CB00001F		LGP11b	EY396521	Predicted: heat shock cognate 70 kDa protein-like [*Brachypodium distachyon*]
7	CB00006F		LGP10	EY396591	Predicted: phosphoenolpyruvate/phosphate translocator 2, chloroplastic [*Vitis vinifera*]
8	CB00055F	LGD10b	LGP10	EY396468	GST6 protein [*Elaeis guineensis*]
9	CB00142	LGD3		JK629438	Pathogenesis-related protein 10c [*Elaeis guineensis*]
10	CB00145	LGD8b		JK629439	Hypersensitive-induced response protein [*Carica papaya*]
11	CEO02026		LGP12c	EY398261	Hypothetical protein SORBIDRAFT_09g002030 [*Sorghum bicolor*]
12	CEO02683	LGD9		EY397095	Sucrose synthase1 [*Elaeis guineensis*]
13	EO02487		LGP10	EY408525	Pathogenesis-related protein [*Elaeis guineensis*]
14	EO02817		LGP8b	EY410649	Serine/threonine protein phosphatase PP1 [*Medicago truncatula*]
15	FDA00089	LGD11c	LGP11b	JK629440	No significant similarity
16	FDB00046	LGD14a		Failed to sequence	–
17	FDB00074		LGP6	JK629441	No significant similarity
18	FDB00086		LGP3	JK629442	No significant similarity
19	FDB00120		LGP1	JK629443	No significant similarity
20	G00016		LGP6	JK629444	Ribosomal protein L32 [*Elaeis guineensis*]
21	G00037	LGD8b		GH159168	No significant similarity
22	G00057		LGP2b	JK629445	Glyceraldehyde 3-phosphate dehydrogenase [*Elaeis guineensis*]
23	G00058		LGP13	JK629446	Predicted: probable polygalacturonase-like [*Vitis vinifera*]
24	G00069	LGD12a	LGP12a	JK629447	Os01g0300200 [*Oryza sativa* Japonica Group].
25	G00080		LGP10	JK629448	Beta-mannosidase 1 [*Oncidium* Gower Ramsey]
26	G00122	LGD11b		JK629449	Hypothetical protein SORBIDRAFT_01g017570 [*Sorghum bicolor*]
27	G00132	LGD13	LGP13	JK629450	No significant similarity
28	G00138A		LGP11a	JK629451	Ubiquitin-conjugating enzyme E2, putative [*Ricinus communis*]
29	G00142	LGD12b	LGP12b	GH159171	No significant similarity
30	G00146		LGP11b	JK629452	Putative DIM-like protein [*Glycine max*]
31	G00152		LGP4a	JK629453	OMT4 [*Vanilla planifolia*]
32	G00158		LGP6	JK629454	Hypothetical protein VITISV_030281 [*Vitis vinifera*]
33	G00163	LGD16	LGP16b	JK629455	40S ribosomal protein S23 [*Elaeis guineensis*]
34	G00170	LGD4b		JK629456	S-adenosylmethionine synthetase 1 [*Oryza sativa* Indica Group]
35	G00200		LGP12a	JK629457	Translationally controlled tumor protein [*Elaeis guineensis*]
36	G00233		LGP4b	JK629458	Chain A, crystal structure of highly glycosylated peroxidase from royal palm [*Roystonea regia*]
37	G00246		LGP8b	JK629459	Ubiquitin conjugating enzyme [*Cicer arietinum*]
38	GT00008	LGD12b	LGP12b	GH159173	No significant similarity
39	K00007	LGD10a	LGP10	JK629460	Ras-related protein RIC1 [*Elaeis guineensis*]
40	K00032A		LGP6	JK629461	Predicted: Low quality protein: polyadenylate-binding protein 3 [*Vitis vinifera*]
41	KT00015	LGD14b		JK629462	Hypothetical protein SORBIDRAFT_02g028940 [*Sorghum bicolor*]
42	KT00029		LGP8b	JK629463	Predicted: universal stress protein A-like protein [*Vitis vinifera*]
43	KT00040	LGD11c	LGP11b	JK629464	Endochitinase precursor (EC 3.2.1.14) [*Nicotiana tabacum*]
44	M00013A	LGD2		JK629465	No significant similarity
45	M00020A		LGP14	JK629466	No significant similarity
46	ME00051		LGP10	JK629467	No significant similarity
47	MET00004		LGP8b	JK629468	Metallothionein-like protein [*Elaeis guineensis*]
48	MT00002		LGP2b	JK629469	Putative cytochrome c oxidase subunit 6b-1 [*Oryza sativa* Japonica Group]
49	MT00030	LGD5	LGP5a	JK629470	No significant similarity
50	MT00045		LGP15	JK629471	No significant similarity
51	MT00060	LGD14a		JK629472	Predicted: Uncharacterized protein LOC100253066 isoform 2 [*Vitis vinifera*]
52	MT00137	LGD13		JK629473	Predicted: Histone H2A-like [*Glycine max*]
53	MT00142		LGP8b	JK629474	No significant similarity
54	RD00049		LGP3	JK629475	Pathogenesis-related protein 10c [*Elaeis guineensis*]
55	SFB00003	LGD4b		JK629476	No significant similarity
56	SFB00012		LGP5b	JK629477	No significant similarity
57	SFB00015		LGP12a	JK629478	Translationally controlled tumor protein [*Elaeis guineensis*]
58	SFB00016	LGD8b	LGP8b	JK629479	No significant similarity
59	SFB00021		LGP5b	GH159184	No significant similarity
60	SFB00022		LGP12a	JK629480	No significant similarity
61	SFB00031		LGP8b	GH159186	Profilin 2 [*Elaeis guineensis*]
62	SFB00039		LGP5b	GH159189	No significant similarity
63	SFB00041	LGD1		GH159190	No significant similarity
64	SFB00042	LGD11c		JK629481	SK3-type dehydrin [*Musa* ABB Group]
65	SFB00043		LGP6	JK629482	No significant similarity
66	SFB00047		LGP15	JK629483	Cationic peroxidase 2 [*Glycine max*]
67	SFB00054	LGD12b	LGP12b	GH159191	Pectinesterase family protein [*Arabidopsis lyrata* subsp. lyrata]
68	SFB00062		LGP2a	GH159193	Hypothetical protein ARALYDRAFT_899257 [*Arabidopsis lyrata* subsp. lyrata]
69	SFB00063	LGD11a	LGP11a	JK629484	Predicted: 60S ribosomal protein L8 [*Vitis vinifera*]
70	SFB00066		LGP12a	JK629485	Predicted: 60S ribosomal protein L8 [*Vitis vinifera*]
71	SFB00072		LGP16b	JK629486	No significant similarity
72	SFB00073		LGP11a	JK629487	Hypothetical protein SORBIDRAFT_06g018700 [*Sorghum bicolor*]
73	SFB00082		LGP4a	JK629488	Ribosomal protein S27 [*Arabidopsis lyrata* subsp. lyrata]
74	SFB00088		LGP12c	JK629489	Metallothionein type 2a-FL [*Elaeis guineensis*]
75	SFB00093	LGD15		JK629490	Hypothetical protein SORBIDRAFT_10g028130 [*Sorghum bicolor*]
76	SFB00097		LGP11a	JK629491	Hypothetical protein SORBIDRAFT_06g018700 [*Sorghum bicolor*]
77	SFB00109	LGD14b		JK629492	No significant similarity
78	SFB00111		LGP2a	JK629493	No significant similarity
79	SFB00118		LGP3	JK629494	Histone H4 [*Zea mays*]
80	SFB00120	LGD2	LGP2a	JK629495	Predicted: pectinesterase inhibitor [*Vitis vinifera*]
81	SFB00130		LGP3	GH159198	No significant similarity
82	SFB00131	LGD4b		JK629496	Ubiquitin [*Morus bombycis*]
83	SFB00141	LGD15		JK629497	No significant similarity
84	SFB00144		LGP11b	JK629498	Putative DIM-like protein [*Glycine max*]
85	SFB00145	LGD10b		JK629499	No significant similarity
86	SFB00152		LGP3	JK629500	Metallothionein-like protein [*Typha latifolia*]
87	SFB00154	LGD14a		JK629501	Ubiquitin extension protein-like protein [*Elaeis guineensis*]
88	SFB00157	LGD15		JK629502	Histone H2B [*Arabidopsis thaliana*]
89	SFB00167		LGP12c	JK629503	Metallothionein-like protein [*Typha latifolia*]
90	SFB00219	LGD8b		JK629504	Ribosomal protein L35A [*Elaeis guineensis*]
91	SFB00241		LGP2a	JK629505	Histone H4 [*Arabidopsis thaliana*]
92	SFB00243		LGP12c	JK629506	No significant similarity
93	SFB00246	LGD12b	LGP12b	JK629507	Histone H2A [*Camellia sinensis*]

Putative IDs were deduced for the SSR-containing sequences by comparing to the non-redundant protein database of GenBank (Blastx). A threshold score of >80 was used to assign significant similarity.

a Two RFLP markers were mapped.

The individual linkage groups were linked to the map published by [14] using mEgCIR markers. For example, mEgCIR0268, mEgCIR3813 and mEgCIR3809 from LG1 in [14] were also mapped in ENL48 and the linkage group thus labeled LGD1 (where ‘LG’ represents the linkage group and ‘D’ *dura*). In some cases, markers reported by [14] to be in one linkage group were separated in ENL48. For example, markers mEgCIR3693, mEgCIR3477 and mEgCIR3557 were mapped in LG4 [14] however, in this study marker mEgCIR3693 was in a separate group from mEgCIR3477 and mEgCIR3557. In this scenario, the two linkage groups were considered as two sub-groups for LGD4 and labeled LGD4a and LGD4b. The resulting framework map covered a total genetic distance of 798.0 cM with an average of 5.4 cM between markers.

As for ML161, of the 608 markers analyzed, 27 were ungrouped and 341 could not be positioned confidently on the map. The remaining 50 AFLPs, 71 RFLPs and 119 SSRs were assigned to 24 groups. In comparison with ENL48, a denser map was constructed for ML161 with 240 markers spanning a total map length of 1,328.6 cM at an average density of 5.5 cM between markers. Similar to ENL48, the linkage groups were labeled accordingly with ‘P’ representing *pisifera*. Stability of the marker order was shown by the co-linearity of the mEgCIR markers compared to those of [14]. Sixteen linkage groups, which also represent the basic numbers of chromosome pairs in oil palm, were identified and labeled LGP1 to LGP16.

The resulting ML161 map was used as second reference map for the ENL48 map by using the co-segregating SSR (from MPOB database) and RFLP markers. This was particularly useful for linking groups between the two parental maps, especially for those that did not have any or with only one mEgCIR marker, such as LGD3, D4a, D5, D7, D10a, D10b, D11a, D11c, D12b, D13, D15 and D16. Using this approach, the alignment of linkage groups between the two parental maps was determined and presented in Figure 2, making comparisons of the positions of markers on the corresponding linkage groups in ENL48 and ML161 much easier.

A total of 53 co-segregating markers (16 RFLPs and 37 SSRs) were mapped on both the ENL48 and ML161 maps. Theoretically, map integration is possible with at least 2 common co-segregating markers in a group. This would indicate that most of the groups in the two parental maps (D1/P1, D2/P2a, D3/P3, D4a/P4a, D4b/P4b, D5/P5a, D7/P7, D8b/P8b, D10a/P10, D11a/P11a, D11c/P11b, D12b/P12b, D13/P13, D15/P15 and D16/P16b) could be integrated. However, our experience in this study was that the numbers of co-segregating markers were not sufficient to accurately combine the two parental maps. It was observed that in almost all the integrated groups (data not shown), the differences in recombination frequencies between the parents were high (0.3–0.5). This could be due to the markers being sparse in one of the parental linkage groups (in this case, mostly on the ENL48 map).

### QTL Associated with Tissue Culture Response

**Figure 3:**
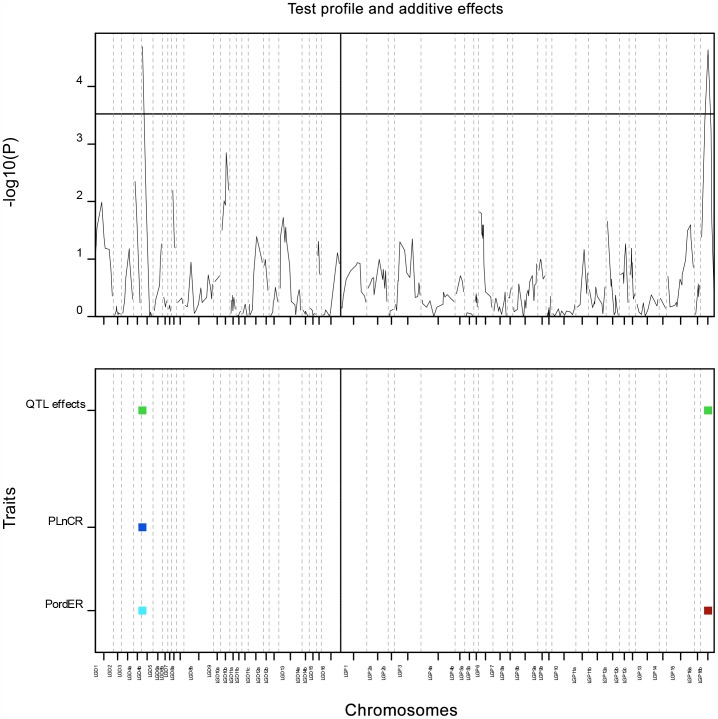
QTLs detected for PLnCR and PordER using Multi-trait QTL analysis, GenStat 14. Upper panel shows the QTL profiles at –log10 (P-value) which resulted from interval mapping scanning. The horizontal line shows the genome-wide significant threshold determined by Li and Ji (P = 3.5). Lower panel shows the QTL effects (green square) resulting from multi-trait interactions: QTL on LGD4b was affected by PLnCR (dark blue square) and PordER (light blue square) while; QTL on LGP16b only contains effect from PordER (brown square).

Two QTLs were detected: each for callogenesis (PLnCR) and embryogenesis (PordER). As shown in Figure 3, a QTL for PLnCR was detected in LGD4b of ENL48 at position zero. The marker pointing to the QTL was mEgCIR3477 and explained 17.5% of the variance; the allele substitution effect was 0.048 (S.E = 0.012). The marker was also found to have a minor effect (0.014±0.006) on PordER explaining 5.7% of the variance. A much more important QTL effect for PordER was detected in LGP16b in ML161. The QTL was located at 26.34 cM (marker sMo00109) and explained 20.1% of the variance; the allele substitution effect was 0.027 (S.E = 0.006).

## Discussion

### Mapping Population

Crosses involving *dura* and *pisifera* palms produce the hybrid *tenera*s which are currently used as commercial planting materials. Therefore, they are of utmost importance to the oil palm industry. The mapping population used in this study was an already existing population involving a cross between Ulu Remis Deli *dura* (ENL48) and Yangambi *pisifera* (ML161). Deli *dura* palms with Ulu Remis genetic background are known to combine well with Yangambi *pisifera*s producing high yielding *tenera* progenies [31]. A few palms from this cross had been tissue cultured before and showed variation in response to tissue culture. As such, this cross of high yielding *tenera*s was deemed appropriate for detecting the QTL for tissue culture amenity.

### Tissue Culture of the Mapping Population

The height of oil palm makes sampling of its young leaves for culture a challenging task. The process requires skilled workers to climb the palm and harvest the very young spear leaves, which have not yet even emerged, without damaging the apical growing point. Because of the sustained damage, repeat sampling of a palm is only possible after three to five years [32]. Thus, re-sampling of palms was not possible within the time frame of the research project. The parental palms were not sampled as they were being actively used in the breeding program and it was not practical to wait for at least three years for the palms to recover. Furthermore, there was a desire to avoid risk of any permanent damage to the palms.

Most of the palms were recalcitrant to tissue culture as was to be expected from previous experience on oil palm worldwide. Significant deviation of tissue culture amenity data from normal distribution had also been frequently reported for other crops, such as red clover [7], wheat [11], rice [10], barley [12] and loblolly pine [33], and the data had to be transformed for normality. Indeed, normality may not be obtained even after transformation, such as in the case of the data on shoot differentiation rate in barley [34], callus formation in maize [35] and callus induction and somatic embryogenesis in rye [36]. As such, in these studies, the non-normal data were used for QTL analysis. In the current study, we improved the normality of CR and ER in two stages (as described in *Materials and Methods*) involving transformations and correction of experimental variables prior to QTL analysis.

### Development of Additional SSR Markers

The SSRs were developed from both ESTs and genomic libraries of oil palm. Mining of these SSRs was previously reported by [17]–[20], [37]. The authors (except [17]) selected some SSRs for genetic diversity studies and this study reports on their applicability to genetic mapping and QTL analysis. Although a large number of SSRs have been reported from the existing oil palm sequence collections, this number is expected to increase rapidly from the genome sequencing project being carried out for oil palm [38]. There is also no doubt that with time, a large number of single nucleotide polymorphism (SNP) markers will also become available for oil palm.

The additional co-segregating SSR markers used in this study are crucial for further saturating and integrating both parental maps. The approach taken was to focus on SSRs rather than RFLPs which are known to be of low throughput and costly. EST-derived SSRs are essentially similar to cDNA RFLP-probes as they are also from the genic regions. The approach was thus appropriate as the EST-SSRs revealed more co-segregating markers (about 38.0%) than the 24.4% obtained by using RFLPs. Previously [14] had shown the potential use of genomic-SSR markers for integrating the *tenera* and *dura* maps. In this study, genomic SSRs were also used and contributed a reasonable number of co-segregating markers - about 35.0% of the total genomic SSRs genotyped. Therefore, there is potential in using both EST- and genomic-derived SSRs for map saturation and integration as observed in this study.

### Oil Palm Genetic Linkage Maps

The current maps were constructed using very stringent parameters (as described in *Materials and Methods*). Markers (mostly AFLPs) as reported in [13] that failed to meet the criteria were excluded from analysis. Removing them resulted in some groups reported earlier, such as group 3 in ML161, to be separated into two sub-groups now labeled as P4a and P4b. Similar changes were observed on groups 7 (now labeled as sub-groups P2a and P2b), 10 (sub-groups P5a and P5b) and 15 (sub-groups P11a and P11b). Significant changes were also observed in groups 1 and 2 of [13] which were separated into 3–4 sub-groups. However, most of the groups - 4, 5, 6, 8, 9, 11, 12, 13 and 16 - remained intact and were renamed LGP1, P10, P13, P7, P15, P12b, P16a, P14 and P16b, respectively, in line with [14]. Changes were also obvious on the ENL48 map. Although the current ENL48 and ML161 maps have more groups, they are greatly improved in accuracy of marker order.

The mapping of published SSR markers (mEgCIR) allowed comparison with a published oil palm genetic map. This, in turn, allowed labeling of linkage groups in the current map to match those by [14]. More importantly, by comparing with the 16 linkage groups reported by [14], linkage groups belonging to the same chromosome could be identified. The orders of common markers were also compared and found to be consistent, boosting confidence in the genetic maps constructed in this study. This also allowed for standardized labeling of every linkage group in both ENL48 and ML161 which also made comparison between the two parental maps much easier.

The genome size for *E. guineensis* is estimated to be 2C = 3.86±0.26 pg [39] which is equivalent to 1,887.54±127 Mbp (number of base pairs = mass in pg×0.978×10^9^, where 1.0 pg = 978 Mbp [40]). Considering the estimates as reference, the ML161 map (1,328.6 cM) has 70.4% genome coverage and ENL48 (798 cM) 42.3%. The estimated genome coverage appears consistent with the marker density observed in the two parental maps. Gaps of >20.0 cM were still observed between markers in the same chromosome. Additional SSR markers (and perhaps SNPs) are needed to saturate the two parental maps. This is also useful to further reduce the number of linkage groups to the basic chromosome number of 16. This would be particularly challenging for ENL48 because its genome appears more homozygous than that of ML161. In fact, the *dura* parent was about 28.0% less polymorphic than the *pisifera*. This is probably due to the narrow genetic background of ENL48 which is a Deli *dura*. In general, the Deli *dura* materials are known to demonstrate less diversity compared to other sources of *E. guineensis* [19]. Furthermore, in oil palm breeding programs, the maternal *dura* lines undergo both selfing and sibbing to increase homozygosity before being crossed with the paternal *pisifera* palm. As such, it is not surprising that ENL48 was more homozygous than the paternal palm (ML161). Therefore, a larger number of SSRs and possibly SNPs have to be screened to saturate the ENL48 map.

### QTLs Associated with Callusing and Embryogenesis Rates

In this study, the numbers of QTLs detected for tissue culture response are within the range reported for rice, barley, wheat, maize, sunflower, *Arabidopsis*, broccoli, poplar and tomato [41]. The type and size of the mapping population are among the factors believed to influence the numbers and effects of the detected QTLs. Ideally, a cross between two palms showing extreme differences in tissue culturability would be more effective for detection of QTL related to tissue culture response. However issues, particularly regarding the availability of palm materials, limited our options in selecting the mapping population to study. With respect to the size of the mapping population, the difficulty in tissue culturing oil palm would not have allowed for too many palms to be used. The 87 palms used in this study already tested the limits of the tissue culture laboratories involved.

The existing tissue culture laboratories in Malaysia do routinely culture oil palm. The numbers of palms and the different genotypes cultured may allow for association analysis of markers to tissue culturability. This may allow for validation of existing markers linked to the QTLs for CR and ER and/or allow detection of additional QTLs. However, the standardization of phenotype data collection and effect of the different media used by the various laboratories on CR and ER will have to be sorted out before this is possible.

It has been suggested that only a few simply inherited genes are of major importance in the genetics of embryogenesis [35]. In oil palm, research had also been carried out on gene expression during embryogenesis. In fact, some interesting genes, such as lipid transfer proteins, were found to be highly expressed in oil palm embryogenic tissues [17]. In other crops, auxin- and wound-responsive genes, such as DNA-binding proteins, calcium-modulated proteins, cell cycle-associated genes, cell wall proteins and glutathione-S-transferase, have also been associated with tissue culture [42]. Therefore, it will be worthwhile to explore some of the identified candidate genes for mapping on the current maps. It will be interesting to see if the candidate markers can be mapped closer to the existing QTLs or can detect additional QTLs.

### Application of QTL in Improving Oil Palm Tissue Culture

Ideally the marker-QTL should be evaluated in other independent crosses of oil palm. This had been done for barley with common QTLs associated with callus growth detected across four populations by [12]. Although it will be a challenging endeavor, the markers linked to QTLs in this study can be used to determine if they reveal the same QTLs in other oil palm populations.

Subject to confirmation of the QTLs in other mapping populations or genotypes, they could be important for selecting ortets to be cultured. Unlike expressed traits (e.g. yield and height), tissue culture amenity remains unknown until the palms are actually cloned. Furthermore, some high yielding palms have at times failed to be cultured. The availability of markers linked to tissue culturability can facilitate the cloning of such palms where, the favorable alleles can be incorporated into the progenies of these palms through marker assisted selection (MAS), and the progenies then cloned. As the markers for yield are becoming available for oil palm [43], it is possible to select palms that are not only high yielding but amenable to tissue culture as well. In fact, the large MPOB oil palm germplasm could be screened for favorable alleles for yield and tissue culture before any palms are included in the breeding program. [6] opined that the biggest advantage of a clone is the early exploitation of new genetic materials produced by introgressing useful gene from wide crosses, which would also help to broaden the genetic base of the current planting materials.

Although the production of oil palm clones has increased, this has more to do with more laboratories entering the fray than a real improvement of the tissue culture process [6]. As such, there remains the need to improve the process to at least allow more of the demand to be satisfied. The markers linked to QTLs for tissue culturability may be helpful in this effort. Palms identified for cloning (based on favorable traits, like high yield or disease resistance), could first be screened with markers to find out whether they have the favorable alleles for tissue culturability. This could help to reduce the time and other resources wasted on tissue culturing recalcitrant palms.

## Supporting Information

Figure S1 **Sampling of unopened spear leaves and the explants used for tissue culture in oil palm.** Figures A & B show the skilled workers climbing the palm to cut the unopened spear leaves from the apical growing point; C: Outer layers of the leaf cabbage are removed except the petioles of frond number 0. This is followed by a longitudinal cut to disclose the internal fronds (fronds −3 to −7 or lower) comprising stacks of young leaflets. D. The leaflets are cut into 12 segments, each having a width of 1.5 cm and sterilized before being cultured on the modified Murashige and Skoog media [15].
(TIF)

Figure S2 **The distribution of phenotypic data for callusing rate (CR) and embryogenesis rate (ER) in Treatments 1 and 2.** The normality in CR1 and CR2 was improved by ln(CR +0.2) transformation and by obtaining a set of predicted data (PLnCR). This was done after removal of experimental variance effects that was generated by the REML variance components analysis. For ER, two transformations were used: (1) a transformation into a binary variable, denoted as binER, with values: 0 if ER = 0 and 1 if ER >0, (2) a transformation into an ordinal variable, denoted as ordER, with three values: 0 if ER = 0, 1 if 0< ER ≤1 and 2 if ER >1. Predictions of the random effects were denoted as PbinER and PordER, respectively.
(TIF)

Table S1 **The profiles of alleles segregating in the P2 mapping population.**
(DOC)

Table S2 **Data obtained from the various markers tested and mapped in the P2 parental linkage maps.**
(DOC)
