# Supplementary figures and images for: Identification of QTLs Associated with Callogenesis and Embryogenesis in Oil Palm Using Genetic Linkage Maps Improved with SSR Markers
Source: PLoS One. 2013 Jan 29;8(1):e53076. doi: 10.1371/journal.pone.0053076 (PMC3558468; doi:10.1371/journal.pone.0053076)

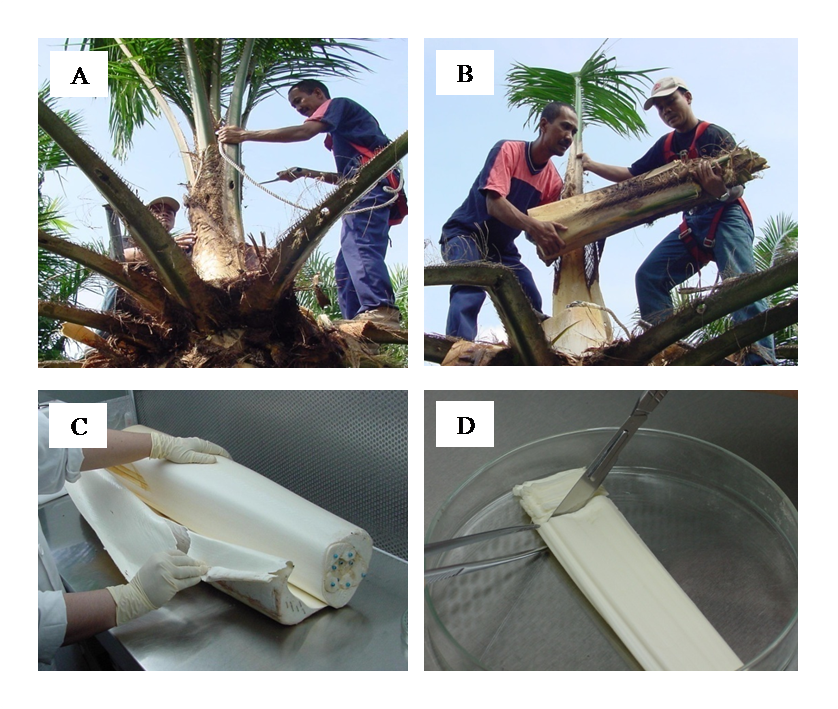

Supplement: Figure S1 — Sampling of unopened spear leaves and the explants used for tissue culture in oil palm. Figures A & B show the skilled workers climbing the palm to cut the unopened spear leaves from the apical growing point; C: Outer layers of the leaf cabbage are removed except the petioles of frond number 0. This is followed by a longitudinal cut to disclose the internal fronds (fronds −3 to −7 or lower) comprising stacks of young leaflets. D. The leaflets are cut into 12 segments, each having a width of 1.5 cm and sterilized before being cultured on the modified Murashige and Skoog media [15]. (TIF) [file pone.0053076.s001.tif]

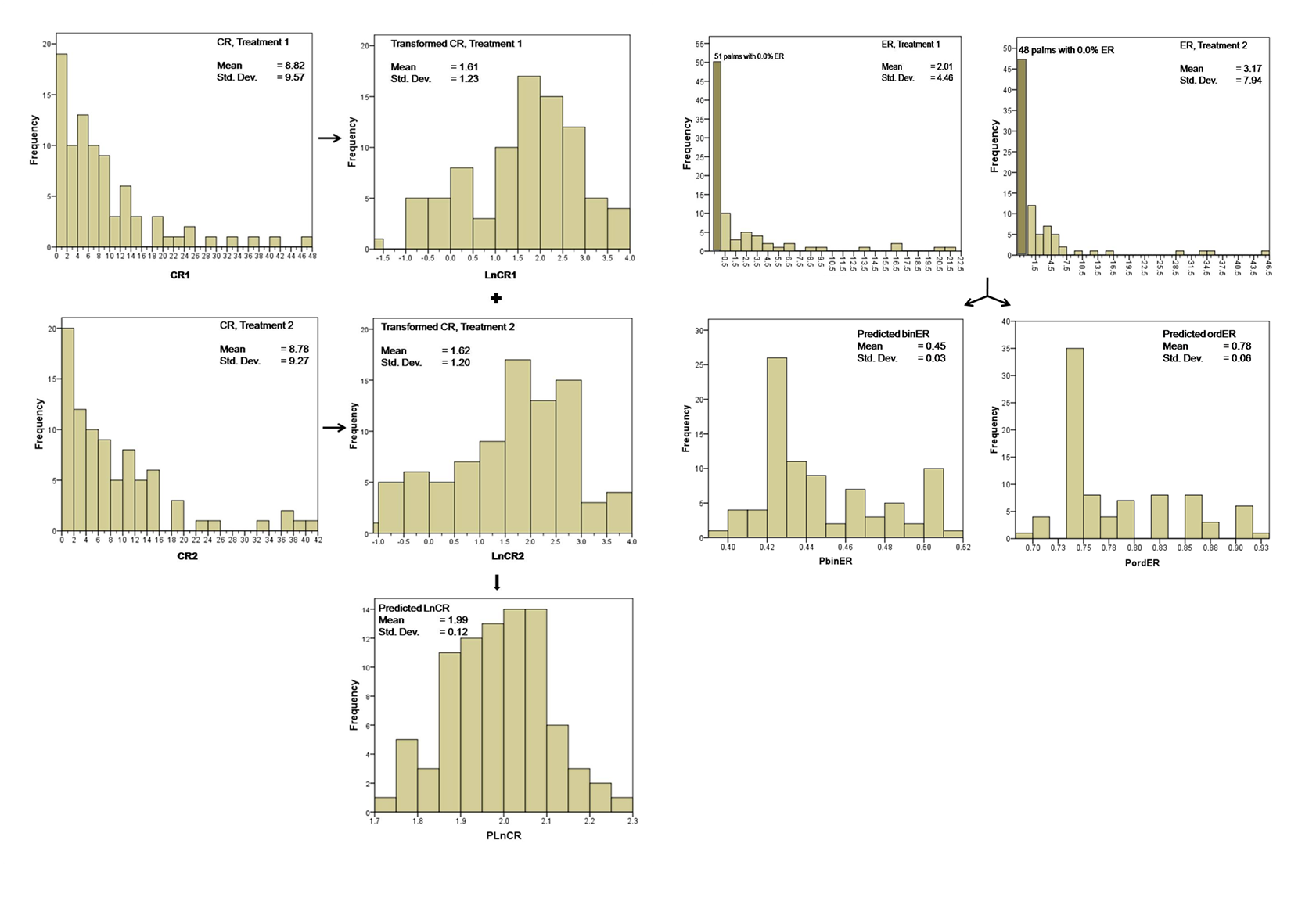

Supplement: Figure S2 — The distribution of phenotypic data for callusing rate (CR) and embryogenesis rate (ER) in Treatments 1 and 2. The normality in CR1 and CR2 was improved by ln(CR +0.2) transformation and by obtaining a set of predicted data (PLnCR). This was done after removal of experimental variance effects that was generated by the REML variance components analysis. For ER, two transformations were used: (1) a transformation into a binary variable, denoted as binER, with values: 0 if ER = 0 and 1 if ER >0, (2) a transformation into an ordinal variable, denoted as ordER, with three values: 0 if ER = 0, 1 if 0< ER ≤1 and 2 if ER >1. Predictions of the random effects were denoted as PbinER and PordER, respectively. (TIF) [file pone.0053076.s002.tif]
