# Supplementary material for: Identification of QTLs Associated with Callogenesis and Embryogenesis in Oil Palm Using Genetic Linkage Maps Improved with SSR Markers
Source: PLoS One. 2013 Jan 29;8(1):e53076. doi: 10.1371/journal.pone.0053076 (PMC3558468; doi:10.1371/journal.pone.0053076)
Supplement: Table S1 — The profiles of alleles segregating in the P2 mapping population. (DOC) [file pone.0053076.s003.doc]

| Profiles by [14] | Segregating alleles | Scoring of alleles | Genotypes observed  in parental palms | | Genotypes observed in progenies | | | | Expected  segregation ratio |
| --- | --- | --- | --- | --- | --- | --- | --- | --- | --- |
|  |  |  | ENL48 | ML161 |  |  |  |  |  |
| 1 | 1 | *l*  *m* | ─  ─ | ─ | ─  ─ | ─ |  |  | 1:1 |
|  |  |  | *lm* | *ll* | *lm* | *ll* |  |  | *lm*:*ll* |
| 1 | 1 | *n*  *p* | ─ | ─  ─ | ─  ─ | ─ |  |  | 1:1 |
|  |  |  | *nn* | *np* | *np* | *nn* |  |  | *nn*:*np* |
| 5 | 2 | *h*  *k* | ─  ─ | ─  ─ | ─ | ─  ─ | ─ |  | 1:2:1 |
|  |  |  | *hk* | *hk* | *hh* | *hk* | *kk* |  | *hh*:*hk*:*kk* |
| 8 | 3 | *e*  *f*  *g* | ─  ─ | ─  ─ | ─ | ─  ─ | ─  ─ | ─  ─ | 1:1:1:1 |
|  |  |  | *ef* | *eg* | *ee* | *ef* | *eg* | *fg* | *ee*:*ef*:*eg*:*fg* |
| 9 | 4 | *a*  *b*  *c*  *d* | ─  ─ | ─  ─ | ─  ─ | ─  ─ | ─  ─ | ─  ─ | 1:1:1:1 |
|  |  |  | *ab* | *cd* | *ac* | *bc* | *ad* | *bd* | *ac*:*bc*:*ad*:*bd* |
