# Supplementary material for: Identification of QTLs Associated with Callogenesis and Embryogenesis in Oil Palm Using Genetic Linkage Maps Improved with SSR Markers
Source: PLoS One. 2013 Jan 29;8(1):e53076. doi: 10.1371/journal.pone.0053076 (PMC3558468; doi:10.1371/journal.pone.0053076)
Supplement: Table S2 — Data obtained from the various markers tested and mapped in the P2 parental linkage maps. (DOC) [file pone.0053076.s004.doc]

|  |  | ENL48 | | | ML161 | | | |
| --- | --- | --- | --- | --- | --- | --- | --- | --- |
|  | SSR | AFLP | RFLP | Total | SSR | AFLP | RFLP | Total |
| Number of markers generated | 171 | 152 | 102 | 425 | 265 | 272 | 165 | 702 |
| Number of severely distorted markers or with 10.0 % missing data points and discarded from analysis | 2 | 58 | 7 | 67 | 5 | 83 | 6 | 94 |
| Number of markers discarded during grouping and map ordering | 92 | 61 | 57 | 210 | 141 | 139 | 88 | 368 |
| Number of markers in the final maps | 77 | 33 | 38 | 148 | 119 | 50 | 71 | 240 |
